# Solvent Fractionation and Acetone Precipitation for Crude Saponins from *Eurycoma longifolia* Extract

**DOI:** 10.3390/molecules24071416

**Published:** 2019-04-10

**Authors:** Lee Suan Chua, Cher Haan Lau, Chee Yung Chew, Dawood Ali Salim Dawood

**Affiliations:** 1 Metabolites Profiling Laboratory, Institute of Bioproduct Development, Universiti Teknologi Malaysia, Skudai, Johor Bahru 81310 UTM, Johor, Malaysia; cherhaan@gmail.com (C.H.L.); alisalim883@yahoo.com (D.A.S.D.); 2 Department of Bioprocess and Polymer Engineering, School of Chemical and Energy Engineering, Faculty of Engineering, Universiti Teknologi Malaysia, Skudai, Johor Bahru 81310 UTM, Johor, Malaysia; chee_yung@live.com.my

**Keywords:** *Eurycoma longifolia*, Simaroubaceae, solvent fractionation, acetone precipitation, saponins, LC-DAD-MS/MS.

## Abstract

*Eurycoma longifolia* is a popular folk medicine in South East Asia. This study was focused on saccharide-containing compounds including saponins, mainly because of their medical potentials. Different organic solvents such as ethyl acetate, butanol, and chloroform were used to fractionate the phytochemical groups, which were consequently precipitated in cold acetone. Solvent fractionation was found to increase the total saponin content based on colorimetric assay using vanillin and sulfuric acid. Ethyl acetate fraction and its precipitate were showed to have the highest crude saponins after acetone precipitation. The samples were shown to have anti-proliferative activity comparable with tamoxifen (IC_50_ = 110.6 µg/mL) against human breast cancer cells. The anti-proliferative activities of the samples were significantly improved from crude extract (IC_50_ = 616.3 µg/mL) to ethyl acetate fraction (IC_50_ = 185.4 µg/mL) and its precipitate (IC_50_ = 153.4 µg/mL). LC-DAD-MS/MS analysis revealed that the saccharide-containing compounds such as *m*/*z* 497, 610, 723, 836, and 949 were abundant in the samples, and they could be ionized in negative ion mode. The compounds consisted of 226 amu monomers with UV-absorbing property at 254 nm, and were tentatively identified as formylated hexoses. To conclude, solvent fractionation and acetone precipitation could produce saccharide-containing compounds including saponins with higher anti-proliferative activity than crude extract against MCF-7 cells. This is the first study to use non-toxic solvents for fractionation of bioactive compounds from highly complex plant extract of *E. longifolia*.

## 1. Introduction

*Eurycoma longifolia* has traditionally been used as ethnomedicine by indigenous people from ASEAN (Association of Southeast Asian Nations) countries to treat many illnesses, such as tertian malaria, ulcer, syphilis, gonorrhea, and dysentery, as well as to relieve headache, stomachache, and insect bites [1]. The roots of the plant, which is locally known as Malaysian Ginseng, are popular mainly because of its aphrodisiac effects [2,3,4]. Quassinoids are the most extensively studied phytochemicals from the roots of the plant, mostly C18-20 quassinoids [5]. They are degraded and highly oxygenated triterpenes, which mainly contribute to the bitter taste of plants of the Simaroubaceae family. Saponins are another important class of bioactive phytochemicals, but they are relatively limited in literature for this plant. This could be due to the difficulties in separating and identifying saponins.

Recently, spectrophotometric techniques have been widely applied to estimate total saponin content, based on the method proposed by Hiai et al. [6] for plant samples. This method uses strong acids such as sulfuric acid and perchloric acid to oxidize triterpene saponins and react with vanillin to give a distinctive red-purple colored complex, which can be measured at the visible range of wavelengths ranging from 473 to 560 nm. The reaction is also dependent upon the structure of ring A in triterpenes [7]. Although it is a simple assay to estimate total saponins, specific saponin compounds need to be identified by chromatographic technique. Generally, saponins do not have a chromophore for ultraviolet absorbance in liquid chromatography. Moreover, saponins exhibit low sensitivity in detectors like refractive index and evaporative light scattering, partly due to the restriction in solvent of choice and gradient condition. Although derivatization using 4-bromophenacyl bromide prior to HPLC analysis was proposed by Slacanin et al. [8], Oleszek et al. [9] and Nowacka and Oleszek [10] found that the derivatized standards decomposed in a short period of time (48 h) at room temperature. Therefore, liquid chromatography coupled with mass spectrometry has been the method of choice in the detection and identification of saponins in recent years. Previously, this combined technique has been used to chemically characterize saponins from *Pulsatilla chinensis* [11], *Paris polyphylla* [12], *Chenopodium quinoa* [13], *Tribulus terrestris*, and *Panax ginseng* [14], and *Quillaja saponaria* bark extract [15].

Saponin consists of triterpenoid or steroidal aglycones that are substituted with different number of sugar moieties or organic acids. Saponins are sometimes called glycosaponins, and the unsubstituted aglycones are classified as sapogenins, which are usually nonpolar. Sapogenins could be either triterpenoid (C30) or steroid (C27) aglycones. The hydrophilic sugar moiety and hydrophobic aglycone of saponins make them act as biological detergents. Sawai and Saito [16] reported that plants often accumulate triterpenoids, including steroids in their glycosylated form, saponins. Indeed, glycosylation could stabilize the compounds against thermal degradation during heat processing, and result in greater bioactivities than their aglycones [17].

According to the Malaysian Standard (MS 2409: 2011) [18], the total glycosaponins of the plant could make up more than 40% *w*/*v* in the freeze dried water extract. This yield is significantly higher than that value recorded for *Panax ginseng*, which is only up to 15% of total saponins [19]. Saponins have been associated with various biological activities, such as anti-inflammatory, cholesterol lowering and anti-cancer properties [20,21,22]. Therefore, it is important to investigate saponins in *E. longifolia* extract in order to explain its enthopharmacological properties scientifically.

The present study investigated total saponin content in *E. longifolia* extract, and in its fractions and precipitates. Because of the complex phytochemicals present in the plant extract, liquid–liquid extraction was used to partition the target compounds, using different solvents such as ethyl acetate, butanol, and chloroform. Subsequently, the organic fractions were precipitated in cold acetone to further recover the compounds. Saponins can be precipitated by lowering the dielectric constant of medium using acetone. Therefore, this work compared the estimated total saponin in different organic fractions and their precipitates, as well as highlighting the fragmentation patterns of the identified saccharide-containing compounds from the fractions and precipitates. The anti-proliferative activities of ethyl acetate fraction and precipitate were also examined, using human breast cancer for comparison.

## 2. Results and Discussion

### 2.1. Effect of Ethanol Concentration on Saponin Content

**Figure 1:**
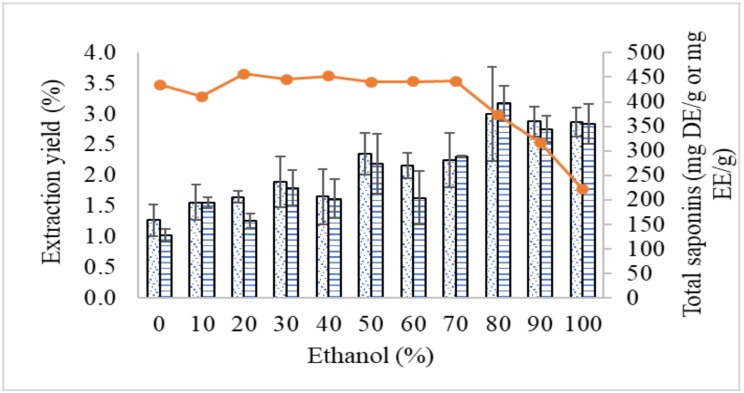
Yield of extraction (line) and total saponins using diosgenin (dot bar) and escin (line bar) as standard chemicals.

Different concentrations of ethanol (0–100%) were used to extract phytochemicals from the roots of *E. longifolia*. In the present study, aqueous ethanol was selected as the solvent of choice, mainly due to the low toxicity and high solubility for terpenes and saponins [23]. The yield of crude extract was around 3.5% using aqueous ethanol ranged from 0–70% (Figure 1). The yield was significantly decreased when the concentration of ethanol was more than 80%. The reduction of solvent polarity by increasing ethanol content showed most saponins in the plant extract to have low solubility. Hence, *E. longifolia* roots might have a high content of polar to semi-polar saponins. The diverse characteristics of saponins are attributed to different functional groups attached to the triterpenoid (pentacyclic structure) or steroidal (tetracyclic structure) aglycones.

The saponin assay showed that the total saponins of *E. longifolia* extracts increased proportionally with the increase of ethanol concentration in the solvent system used for reflux extraction. This also indicates higher solubility of saponins in ethanol than in water. The results expressed as escin equivalent or diosgenin equivalent are almost similar. The ratio of both results is near 1 at different ethanol concentrations. Although diosgenin is not a saponin, the hydroxyl group at C-3 and double bond at C-5 could react with acidic vanillin under oxidization of sulfuric acid to form chromogen. The assay involves the condensation reaction of the aldehyde group of vanillin with the hydroxyl group of triterpenic acid to form red condensates for detection.

### 2.2. Fractionation by Liquid–Liquid Extraction

**Figure 2:**
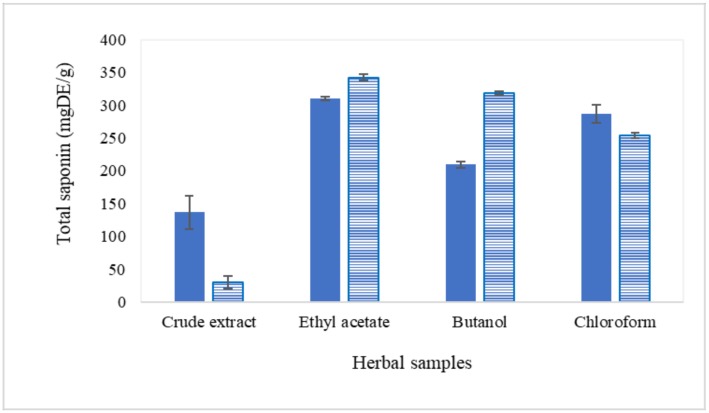
Total saponins of organic fractions (solid bar) and precipitates (line bar) expressed as mg DE/g in different solvent systems.

Organic solvents such as ethyl acetate, butanol, and chloroform were used to partition crude extract using the technique of liquid–liquid extraction. The highly polar substances, such as organic acid, polysaccharides, and proteins, may stay in the aqueous phase, while the other relatively less polar compounds, including terpenoids and saponins, are partitioned into the organic phase. This explains the increment of total saponin content after fractionation (Figure 2). Ethyl acetate fraction was found to have the highest total saponin content among the organic fractions.

**Figure 3:**
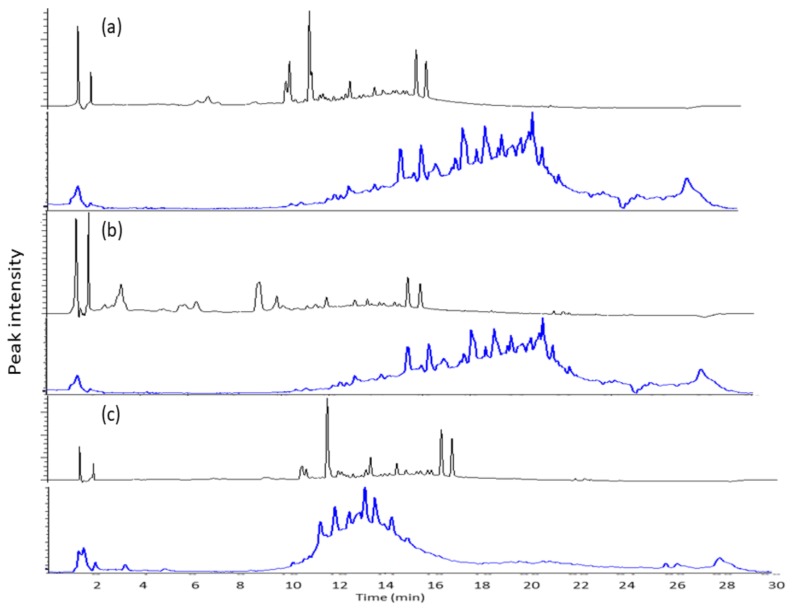
Chromatograms (black line) and total ion chromatograms (blue line) of mass spectrometer for organic fractions from ethyl acetate (**a**), butanol (**b**), and chloroform (**c**).

The chromatograms shown in Figure 3 show the peaks detected at 254 nm after fractionation. The chromatographic profiles of the organic fractions are almost similar, but their mass spectra are significantly different. The chromatographic profiles display the compounds with UV-absorbing property, whereas compounds like terpenoids and saponins mostly do not have such a property. On the other hand, the mass analyzer will only detect ionizable compounds in the samples. Ethyl acetate and butanol fractions were found to have less polar compounds, whereas chloroform seemed to partition more polar compounds from crude extract. The difference between the organic fractions can also be seen from the results of colorimetric assays. In line with the colorimetric assay, the butanol fraction exhibited the lowest peak area.

### 2.3. Cold Acetone Precipitation

Cold acetone precipitation appeared to slightly increase the total saponin content from the organic fractions. The increment was more significant for butanol fraction. Again, ethyl acetate precipitate could obtain the highest total saponin content. The fractions were added dropwise into cold acetone, and precipitate was formed due to the sudden drop of dielectric constant of media. The dielectric constant (ε) of acetone is 20.7, which is about half the dielectric constant of 70% ethanol (ε = 41.1). Precipitation occurred, most probably because of the steric hindrance of lipophilic terpenoidal skeleton, limiting its solubility in acetone. Usually, glycosylated terpenoids or steroids were precipitated in acetone, thus contributing to higher saponin content.

**Figure 4:**
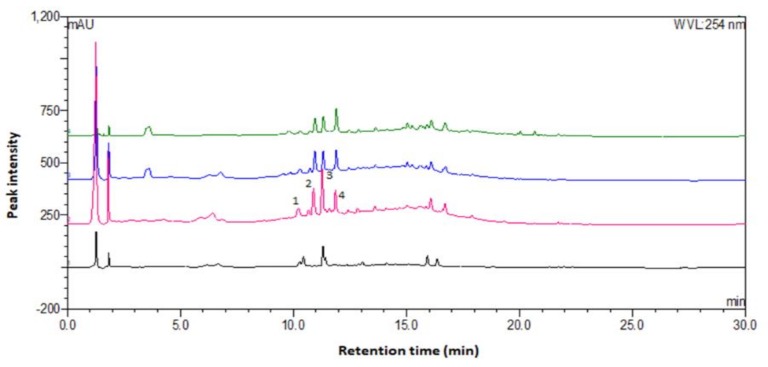
Chromatograms of precipitates in acetone using different organic fractions.

**Figure 5:**
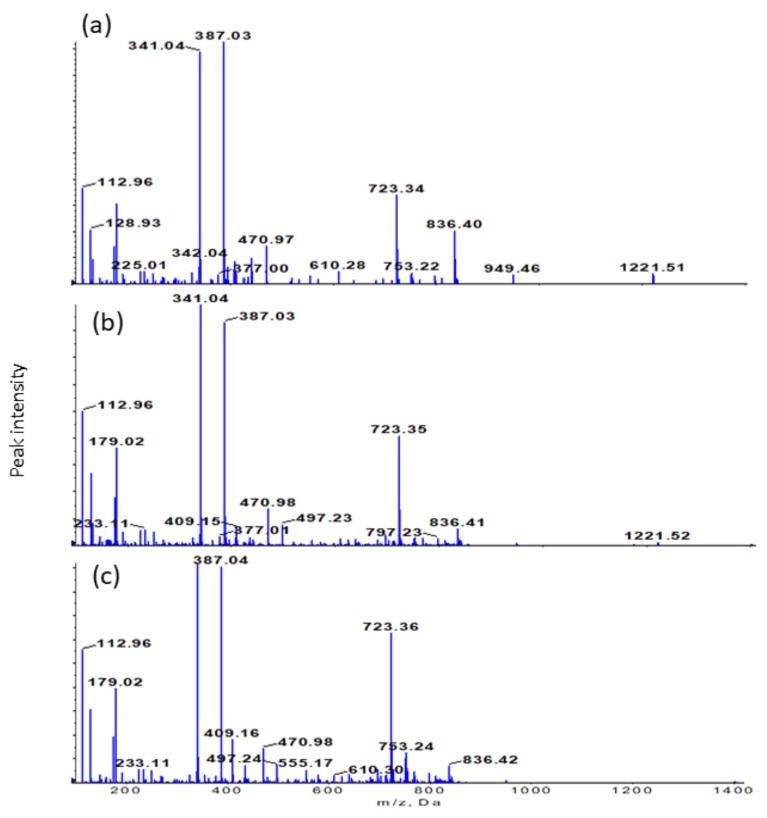
Mass spectra of ethyl acetate (**a**), butanol (**b**), and chloroform (**c**) precipitates.

The chromatograms of the precipitates show peak 1 (10.6 min), peak 2 (10.9 min), peak 3 (11.1 min), and peak 4 (12.2 min), as illustrated in Figure 4. They are tentatively identified as *m*/*z* 427, *m*/*z* 239 (β-carboline-1-propionic acid), *m*/*z* 497, and *m*/*z* 723, respectively. The mass spectra at those retention times revealed the presence of *m*/*z* 497 [2M + HCOOH − H]^−^, *m*/*z* 610 [2M + HCOOH + 113 − H]^−^, *m*/*z* 723 [3M + HCOOH − H]^−^, *m*/*z* 836 [3M + HCOOH + 113 − H]^−^, and *m*/*z* 949 [4M + HCOOH − H]^−^. In the present study, M was found to be the monomer of 226 amu, which is formylated hexose. Most probably, the neutral loss of 113 is acylglycerol. The formylated saccharides have the same fragmentation patterns as the compounds detected in *Rhamnus davurica* Pall [24] and *Rubia cordifolia* L. [25] extracts. Hence, cold acetone was likely to precipitate saccharide-containing compounds, as presented in Table S1 (Supplementary Materials). The table lists the product ions and neutral losses attributed to sugar moieties. The neutral loss of the peaks revealed that the precipitated compounds were saccharide-containing compounds, including saponins. Since saponins do not have chromophores for UV detection, their mass spectra are presented in Figure 5. The mass spectra of the precipitates clearly show the fragment ions, which were mostly ionized saccharides, as intense peaks. The common sugar fragment ions in the figure are *m*/*z* 179 (hexose − H), 225 (hexose + HCOOH − H), 341 (dihexose − H_2_O − H), 377 (dihexose + H_2_O − H), and 387 (dihexose − H_2_O + HCOOH − H). Negative ionization was also found to be more preferable for the precipitated compounds in this study.

**Figure 6:**
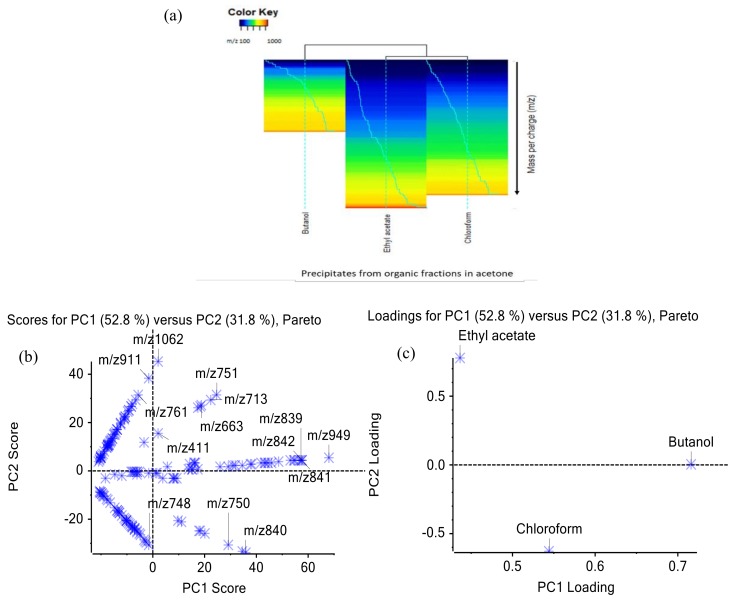
Heat mapping (**a**) and principal component analysis with score (**b**) and loading (**c**) plots.

High performance unsupervised statistical techniques, namely heat mapping and principal component analysis, were used to classify the huge datasets. The heat map explains that the number of metabolites precipitated from butanol fraction was less than the other two organic fractions (Figure 6a). The butanol precipitate contained higher masses of compounds, mostly higher than 600 Da, while the ethyl acetate precipitate was found to have a wide range of compounds. The mass profile of the ethyl acetate precipitate was close to the mass profile of the chloroform precipitate based on the dendogram. In line with the dendrogram, ethyl acetate and chloroform precipitates showed closer metabolite profiles, as explained by the first principal component (PC1) in Figure 6c. The first two principal components explain 84.6% of the total variance for the precipitates. Hence, different organic solvents extracted different metabolites from the crude extract of *E. longifolia*, subsequently contributing to different profiles of metabolites in those precipitates.

### 2.4. Phytopharmacological Significance of Bioactive Precipitate

**Figure 7:**
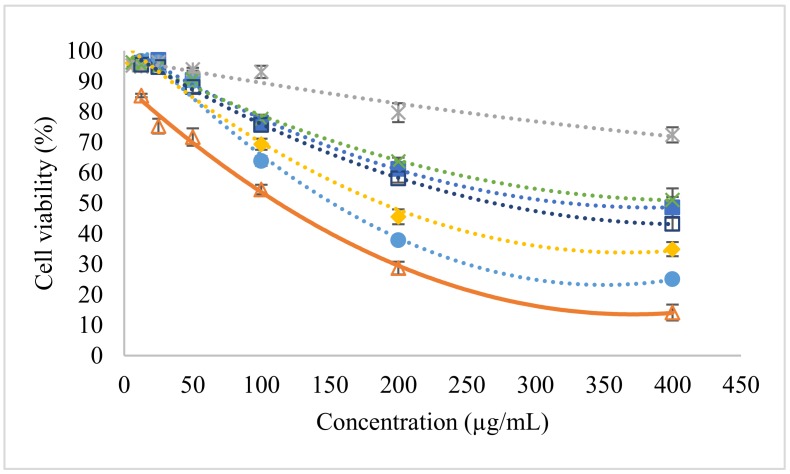
MCF-7 cell viability after treated with tamoxifen (∆), crude extract (■), crude precipitate (x), crude filtrate (□), ethyl acetate fraction (♦), ethyl acetate precipitate (•), and ethyl acetate filtrate (✴).

Ethyl acetate fraction and its precipitate were found to exhibit the highest total saponin content. Therefore, the anti-proliferative activities of ethyl acetate fraction and its precipitate were tested on a human breast cancer cell line in the subsequent experiments. The results found that the inhibitory action of the samples was improved from crude extract (IC_50_ = 616.3 µg/mL) to ethyl acetate fraction (IC_50_ = 185.4 µg/mL) and its precipitate (IC_50_ = 153.4 µg/mL). The IC_50_ of ethyl acetate precipitate was close to the value of tamoxifen, 110.6 µg/mL (Figure 7). Tamoxifen is the most common drug used to treat breast cancer patients [26]. Previous studies also reported that saponins could be potential anticancer agents [21,27]. Hence, the processing technology to concentrate terpenoids and their glycosylated derivatives could increase the performance of herbal samples in suppressing MCF-7 cell proliferation. This can also be seen from the high IC_50_ (733.7 µg/mL) of ethyl acetate filtrate, which means a high concentration of sample is required to exhibit its anti-proliferative activity. Direct precipitation using crude extract seemed to be less cytotoxic against MCF-7 cells. The combination of solvent fractionation and acetone precipitation could increase the biological activity of the plant extract. This technique should be recommended, as previous researchers used toxic solvents such as methanol and chloroform to get bioactive fraction in their pharmacological studies [28,29]. The presence of solvent residues in the plant extracts make them unsuitable for product formulation, particularly products for human consumption. Therefore, food grade solvents such as ethanol, acetone, and ethyl acetate are the primary choice of consumers.

## 3. Materials and Methods

### 3.1. Chemicals and Plant Material

The roots of *Eurycoma longifolia* (SK 3317/18) were harvested from Bentong, Pahang, Malaysia. The samples were then dried and shredded into chip form, about 1 cm in size. Human breast cancer cell line (MCF-7) was obtained from the American Type Culture Collection (ATCC, Manassas, VA, USA) and maintained in Dulbecco’s Modified Eagle Medium. Dimethyl sulfoxide (DMSO, 99.9%), vanillin (≥97%), oleanolic acid (≥97%), and escin (≥95%) were purchased from Sigma-Aldrich, St. Louis, MO, USA. Ethanol, acetic acid, perchloric acid, sulfuric acid, formic acid, ethyl acetate, butanol, chloroform, acetonitrile, and acetone were sourced from Merck, Darmstadt, Germany.

### 3.2. Heat Reflux Extraction

The dried *E. longifolia* chips were then finely ground into powder (~2 mm) by a grinder. The samples (25 g) were extracted with different concentrations of ethanol (250 mL) in a heat reflux system for 2 h. After extraction, the solution was cooled and filtered for drying using a rotary evaporator. The extraction yield was recorded for each solvent system.

### 3.3. Liquid-Liquid Extraction

The crude extract of 70% ethanol was used in the fractionation process. The fractionation was carried out using the technique of liquid–liquid extraction. Three types of organic solvents, namely ethyl acetate, butanol, and chloroform were selected to partition crude extract into individual fractions. Crude extract (0.5 g) was reconstituted in water (10 mL) and vigorously extracted by ethyl acetate (20 mL) in a 100 mL separating funnel. The solution was left for phase separation after extraction. The organic phase was withdrawn, and another 20 mL ethyl acetate was added into the remaining aqueous solution for extraction again. This fractionation process was repeated in triplicate. The organic fraction of ethyl acetate was combined and dried by a rotary evaporator. A similar fractionation process was also carried out to prepare butanol and chloroform fractions.

### 3.4. Cold Acetone Precipitation

The fractions of ethyl acetate, butanol, and chloroform were then reconstituted in 70% ethanol (5 mL) and added dropwise into cold acetone (20 mL). Phytochemicals with poor solubility in cold acetone were precipitated. The precipitated phytochemicals were filtered and dissolved in 50% methanol for LC-DAD-MS/MS analysis, and dissolved in 50% ethanol for total saponin assay.

### 3.5. Total Saponin Content

The total saponin content was determined colorimetrically according to the procedures described by Makkar et al. [30]. A 250 µL sample (1 mg/mL) was mixed with 250 µL vanillin (8g/100 mL ethanol) and topped up with 2.5 mL sulfuric acid (72%). The mixture was heated for 10 min at 60 °C, and then cooled in an ice-water bath for 5 min. The absorbance of the mixture was recorded by a UV-vis spectrophotometer (UV-1800, Shimadzu, Japan) at 544 nm. Escin (5.7–71.4 mg/L) was used as the standard chemical to build a calibration curve. The results are expressed as mg escin equivalent per g sample (mg EE/g), or mg diosgenin equivalent per g sample (mg DE/g).

### 3.6. Cell Proliferation Using MTT Assay

MTT assay was performed to determine the viability of MCF-7 cells treated with samples (crude extract, ethyl acetate fraction, and its precipitate). Standard chemicals, namely escin and tamoxifen were used as positive controls in the experiments, whereas DMSO was used as a negative control. Tumor cells (1 × 10^5^ cell/mL) were seeded in 96 flat well microtiter plates, with 200 μL culture medium in each well. The microplate was covered by sterilized parafilm and shaken gently before incubation at 37 °C, in 5% CO_2_ for 24 h. After incubation, the medium was removed and two-fold serial dilutions of samples were added to the wells for 24 h treatment at 37 °C with 5 % CO_2_. A 10 μL MTT solution was added to each well and further incubated at 37 °C for 4 h. The media solution was carefully removed and 100 μL of solubilization solution was added into each well. The absorbance was determined using an ELISA reader at a wavelength of 575 nm. Each concentration of samples was assayed in triplicate. The growth of MCF-7 cells treated with herbal samples was determined based on their viability after treatment. The results are expressed in effective concentration required to inhibit 50% of viable cells (IC_50_).

### 3.7. LC-DAD-MS/MS

A liquid chromatograph (Dionex Corporation Ultimate 3000; Sunnyvale, CA, USA) integrated with a diode array detector (Dionex Ultimate 3000) and a quadrupole and time-of-flight (QTOF) mass spectrometer (AB SCIEX QSTAR Elite; Foster City, CA, USA) was used to screen phytochemicals. A C18 reversed phase XSelect HSS T3 column (2.1 × 100 mm, 2.5 µm) with a flow rate of 150 µL/min was used for separation, and compound peaks were detected at 254 nm. A binary gradient system consisting of solvent A (water with 0.1% formic acid) and solvent B (acetonitrile) was programmed as: 0–10 min, 10% B; 10–20 min, 10–85% B; 20–25 min, 85% B; 25–25.1 min, 85–10% B; 25.1–30 min, 10% B. The injection volume was 5 µL. All samples were filtered with 0.2 mm nylon membrane filter prior to injection.

The QTOF mass spectrometer was used for phytochemical screening from *m*/*z* 100–2000. A single information dependent acquisition (IDA) method was created to acquire both TOF MS and two dependent runs of product ion scan with rolling collision energy. Nitrogen gas was used for nebulizing (40 psi) and curtain gas (20 psi). Collision gas was set at 3, the accumulation time was 1 s for TOF MS and 2 s for each product ion scan. The voltage of ion spray was 4500 V for negative ion mode. The declustering potential was 40 V and the focusing potential was set at 300 V.

### 3.8. Statistical Analysis

Heat-map and cluster analysis were used to visually classify the detected ions in precipitates using R version 2.11.1. Principal component analysis was carried out using Pareto scaling in the data processing software MarkerView 1.1 (Applied Biosystems/MDSSciex, Foster City, CA, USA).

## 4. Conclusions

The technique of solvent fractionation, followed by acetone precipitation seems to be able to recover saccharide-containing compounds from the highly complex crude extract of *E. longifolia*. Usually, large molecules like triterpenoids and saponins which have intermediate polarity would be recovered and then further precipitated in a highly polar acetone. Ethyl acetate appears to be more effective to recover saccharide-containing compounds. This is the first study to concentrate saccharide-containing compounds for MCF-7 cell inhibition. Further investigation will be carried out to identify the recovered saccharide-containing compounds.

## Supplementary Materials

The following are available online, Table S1: Precipitated compounds in cold acetone from organic fractions of *Eurycoma longifolia*.

## References

[1] Kuo P.C., Damu A.G., Lee K.H., Wu T.S. (2004). Cytotoxic and antimalarial constituents from the roots of *Eurycoma longifolia*. Bioorg. Med. Chem..

[2] Gimlette J.D., Thomson H.W. (1977). A Dictionary of Malayan Medicine.

[3] Ang H.H., Ngai T.H. (2001). Aphrodisiac evaluation in non-copulator male rats after chronic administration of *Eurycoma longifolia* Jack. Fundam. Clin. Pharmacol..

[4] Ang H.H., Ngai T.H., Tan T.H. (2003). Effects of Eurycoma longifolia Jack on sexual qualities in middle aged male rats. Phytomedicine.

[5] Chua L.S., Mohd Amin N.A., Neo J.C.H., Lee T.H., Lee C.T., Sarmidi M.R., Aziz R.A. (2011). LC–MS/MS-based metabolites of *Eurycoma longifolia* (Tongkat Ali) in Malaysia (Perak and Pahang). J. Chromatogr. B.

[6] Hiai S., Oura H., Nakajima T. (1976). Color reaction of some sapogenins and saponins with vanillin and sulfuric acid. Planta Med..

[7] Polonsky J., Runeckles V.C., Mabry T.J. (1973). Chemistry and Biogenesis of the Quassinoids (Simaruobolides). Terpenoids: Structure, Biogenesis, and Distribution: Recent Advances in Phytochemistry.

[8] Slacanin I., Marston A., Hostettmann K. (1988). Quantitative HPLC analysis of molluscicidal saponins from *Phytolacca dodecandra*. Planta Med..

[9] Oleszek W., Price K.R., Colquhoun I.J., Jurzysta M., Ploszynski M., Fenwick G.R. (1990). Isolation and identification of alfalfa (*Medicago sativa* L.) root saponins: Their activity in relation to a fungal bioassay. J. Agric. Food Chem..

[10] Nowacka J., Oleszek W. (1992). High performance liquid chromatography of zanhic acid glycoside in alfalfa (*Medicago sativa*). Phytochem. Anal..

[11] Ouyang H., Guo Y., He M., Zhang J., Huang X., Zhou X., Jiang H., Feng Y., Yang S. (2015). A rapid and sensitive LC-MS/MS method for the determination of Pulsatilla saponin D in rat plasma and its application in a rat pharmacokinetic and bioavailability study. Biomed. Chromatogr..

[12] Yang G., Lu W., Pan M., Zhang C., Zhou Y., Hu P., Hu M., Song G. (2017). An LC–MS/MS method for simultaneous determination of nine steroidal saponins from *Paris polyphylla* var. in rat plasma and its application to pharmacokinetic study. J. Pharm. Biomed. Anal..

[13] Madl T., Sterk H., Mittelbach M., Rechberger G.N. (2006). Tandem mass spectrometric analysis of a complex triterpene saponin mixture of *Chenopodium quinoa*. J. Am. Soc. Mass Spec..

[14] Liu S., Liu M., Liu Z., Song F., Mo W. (2004). Structural analysis of saponins from medicinal herbs using electrospray ionization tandem mass spectrometry. J. Am. Soc. Mass Spec..

[15] Van Setten D.C., Zomer G., van de Werken G., Wiertz E.J.H.J., Leeflang B.R., Kamerling J.P. (2000). Ion trap multiple-stage tandem mass spectrometry as a pre-NMR tool in the structure elucidation of saponins. Phytochem. Anal..

[16] Sawai S., Saito K. (2011). Triterpenoid biosynthesis and engineering in plants. Front. Plant Sci..

[17] Moses T., Papadopoulou K.K., Osbourn A. (2014). Metabolic and functional diversity of saponins, biosynthetic intermediates and semi-synthetic derivatives. Crit. Rev. Biochem. Mol. Biol..

[18] (2011). Phytopharmaceutical Aspect of Freeze Dried Water Extract from Tongkat Ali Roots—Specification.

[19] Hou J.P., Jin Y. (2004). Chapter 5: Miraculous Tonic Herbs: Strengthening the First Line of Defense and Fortifying the Immune System. The Healing Power of Chinese Herbs and Medicinal Recipes.

[20] Xiang L., Yi X., Wang Y., He X. (2016). Antiproliferative and anti-inflammatory polyhydroxylated spirostanol saponins from *Tupistra chinensis*. Sci. Rep..

[21] Xu X.H., Li T., Fong C.M.V., Chen X., Chen X.J., Wang Y.T., Huang M.Q., Lu J.J. (2016). Saponins from Chinese medicines as anticancer agents. Molecules.

[22] Milgate J., Roberts D.C.K. (1995). The nutritional & biological significance of saponins. Nutr. Res..

[23] Gafner S., Bergeron C., McCollom M.M., Cooper L.M., McPhail K.L., Gerwick W.H., Angerhofer C.K. (2004). Evaluation of the efficiency of three different solvent systems to extract triterpene saponins from roots of *Panax quinquefolius* using high-performance liquid chromatography. J. Agric. Food Chem..

[24] Chen G., Li X., Saleri F., Guo M. (2016). Analysis of flavonoids in *Rhamnus davurica* and its antiproliferative activities. Molecules.

[25] Zheng Z., Li S., Zhong Y., Zhan R., Yan Y., Pan H., Yan P. (2017). UPLC-QTOF-MS identification of the chemical constituents in rat plasma and urine after oral administration of *Rubia cordifolia* L. extract. Molecules.

[26] Villegas V.E., Rondón-Lagos M., Annaratone L., Castellano I., Grismaldo A., Sapino A., Zaphiropoulos P.G. (2016). Tamoxifen treatment of breast cancer cells: Impact on Hedgehog/GLI1 signaling. Int. J. Mol. Sci..

[27] Man S., Gao W., Zhang Y., Huang L., Liu C. (2010). Chemical study and medical application of saponins as anti-cancer agents. Fitoterapia.

[28] Tee T.T., Azimahtol H.L.P. (2005). Induction of apoptosis by *Eurycoma longifolia* Jack extracts. Anticancer Res..

[29] Hussain Z., Mohamad I.N., Shuid A.Z. (2019). *Eurycoma longifolia*, a potential phytomedicine for the treatment of cancer: Evidence of p53-mediated apoptosis in cancerous cells. Curr. Drug Targets.

[30] Makkar H.P.S., Siddhuraju P., Becker K. (2007). Saponins, Plant Secondary Metabolites. Methods in Molecular Biology.

