# Supplementary material for: Solvent Fractionation and Acetone Precipitation for Crude Saponins from Eurycoma longifolia Extract
Source: Molecules. 2019 Apr 10;24(7):1416. doi: 10.3390/molecules24071416 (PMC6480193; doi:10.3390/molecules24071416)
Supplement: Supplementary file 1 [file molecules-24-01416-s001.pdf]

Table S1. Precipitated compounds in cold acetone from organic fractions of *Eurycoma longifolia*

Rt (min)	m/z (-)	Fragment ion (-neutral loss)	Putative compound	Ethyl acetate	Butanol	Chloroform
9.6	411	319(-92)/291(-120)/273(-138)/**249(-162)**/224	unknown	√	√	x
10.6	427	343(-84)/**265(-162)**	unknown	√	x	x
10.9	239	221(-18)/195(-44)	β-carboline-1-propionic acid	√	√	√
11.1	497	451(-46)/**225**(-226)	formylated dihexose	x	√	√
11.7	610	564(-46)/546/**451**(-113)/**338/225**(-339)	formylated acylglycerol dihexose	√	√	√
11.9	269	251(-18)/210	unknown	√	√	√
12.2	723	677(-46)/659/593(-84)/470/**451**(-226)/**387**/367/**342**/**338**(-339**)/225/179**	formylated trihexose	√	√	√
12.6	836	790(-46)/**451**(-339)/**338/225**	formylated diacylglycerol dihexose	√	√	√
12.7	949	903(-46)/897/836/**451**(-452)/**225**(-226)	formylated tetrahexose	√	√	√
13.2	643	**463(-180**)/418(-45)/407(-236)/400/306/248(-152)/221/208/**179**/154	unknown	√	√	x
13.5	663	**483(-180)**/438(-225)/407(-256, -31)/326(-112)/221(-217)/154	unknown	√	√	x
14.9	682	**598(-84)/484(-198)**/441/**374(-308)**/**324(-160)**/306/247/225	hydroinositol hexosyl rhamnosyl glucoronide	√	√	√
15.3	702	512(-190)/485(-217)/**374**/**344(-358)**/326/282	unknown	√	√	√
15.8	696	514(-182)/485(-211)/**374**/**338(-358)**/320/238	Hydrocaffeoyl derivative	√	√	√
16.1	639	**414(-225**)/**383(-256, -31**)/317/273/229/180	unknown	√	√	√
16.8	811	**503(-308)/324(-179)**	Pentasaccharide (rutinosyl raffinoside)	√	√	√
19.9	595	**415(-180)**/315/279(-136)/241	Inositol derivatives	x	√	√
20.2	751	**409(-342)**/350/237/191/151	unknown	√	√	x
20.8	753	723/647/597/**555(-198)**/433/365(-190)/387/**341(-214**)/295(-26)/237/179	unknown	√	√	√
23.1	579	**255(-324)**/187	Glucoisoliquiritin	√	√	√
